# The Effects of a Ketogenic Diet on Sensorimotor Function in a Thoracolumbar Mouse Spinal Cord Injury Model

**DOI:** 10.1523/ENEURO.0178-20.2020

**Published:** 2020-08-12

**Authors:** Kyle A. Mayr, Charlie H.T. Kwok, Shane E.A. Eaton, Glen B. Baker, Patrick J. Whelan

**Affiliations:** 1 Hotchkiss Brain Institute, University of Calgary, Calgary, Alberta T2N 1N4, Canada; 2 Department of Neuroscience, University of Calgary, Calgary, Alberta T2N 2T9, Canada; 3 Department of Comparative Biology and Experimental Medicine, University of Calgary, Calgary, Alberta T2N 1N4, Canada; 4 Department of Psychiatry (NRU), Faculty of Medicine and Dentistry, University of Alberta, Edmonton, Alberta T6G 2B7, Canada

**Keywords:** function, ketogenic diet, plasticity, spinal cord injury, therapies

## Abstract

Spinal cord injury and peripheral nerve injuries are traumatic events that greatly impact quality of life. One factor that is being explored throughout patient care is the idea of diet and the role it has on patient outcomes. But the effects of diet following neurotrauma need to be carefully explored in animal models to ensure that they have beneficial effects. The ketogenic diet provides sufficient daily caloric requirements while being potentially neuroprotective and analgesic. In this study, animals were fed a high-fat, low-carbohydrate diet that led to a high concentration of blood ketone that was sustained for as long as the animals were on the diet. Mice fed a ketogenic diet had significantly lower levels of tyrosine and tryptophan, but the levels of other monoamines within the spinal cord remained similar to those of control mice. Mice were fed a standard or ketogenic diet for 7 d before and 28 d following the injury. Our results show that mice hemisected over the T10–T11 vertebrae showed no beneficial effects of being on a ketogenic diet over a 28 d recovery period. Similarly, ligation of the common peroneal and tibial nerve showed no differences between mice fed normal or ketogenic diets. Tests included von Frey, open field, and ladder-rung crossing. We add to existing literature showing protective effects of the ketogenic diet in forelimb injuries by focusing on neurotrauma in the hindlimbs. The results suggest that ketogenic diets need to be assessed based on the type and location of neurotrauma.

## Significance Statement

There is an urgent need for therapeutics to improve outcomes for patients with neurotrauma. Here we test the effects of a noninvasive diet-based therapy. Ketogenic diets, which are high fat and low carbohydrate based, have been shown to be effective in treating epilepsy and Parkinson’s disease, and show promise for treating other neurotrauma and neurodegenerative conditions. Here we show that while we were successful in producing high ketone concentrations in mice, the effects on the recovery of function and pain following a thoracic spinal cord hemisection or spared nerve injury were minimal. Therefore, ketogenic diets, while effective in certain cases, should be evaluated depending on the injury type.

## Introduction

Nerve injuries to the CNS and peripheral nervous system comprise a heterogeneous group of conditions that cause significant disability and also share common characteristics. There are multiple underlying causes contributing to peripheral nerve injury and spinal cord injury (SCI) such as neurotrauma, demyelination, and autoimmune disorders (Ahuja and Fehlings, 2016). In addition to movement disorders, patients with neural injuries have high incidences of persistent pain. Epidemiological data suggest that 53% of SCI patients experience pain (Burke et al., 2017), with the global prevalence of neuropathic pain (i.e., pain as a result of nerve injuries) estimated at 7% (van Hecke et al., 2014). More importantly, persistent pain inhibits patients from adhering to rehabilitative therapies (Turk and Rudy, 1991); thus, pain and loss in motor functions form a negative feedback loop that further perpetuates disabilities (Thompson et al., 2016).

For patients with nerve injuries, improvement in motor functions and reduction in chronic pain are important for quality of life (Adams and Hicks, 2005; Jensen et al., 2007). However, few therapies exist to target both pain and motor functions simultaneously, in part because of the diversity in etiologies and disease progress. Most therapies targeting neurodegeneration and pain are symptomatic and do little to halt further loss in function (Scholz et al., 2009). Existing pain therapies including gabapentin and opioids all have side effects (Teasell et al., 2010), with the development of tolerance and dependence hindering treatment success. Overall, patients rely on combinatorial therapies, requiring multiple types of medications and management, thus resulting in suboptimal rates of adherence to their prescriptions. The identification of a more comprehensive therapy that targets pain and movement disorders is urgently needed for improving care in nerve injuries (Simpson et al., 2012; Teasell et al., 2010).

A key feature of nerve injury is neuroplasticity, which broadly manifests as changes in neuronal excitability and may aid spontaneous recovery by allowing sprouting of neural fibers reinforcing reflex and propriospinal pathways (Bareyre et al., 2004; Finnerup and Baastrup, 2012). However, mechanisms underlying neuroplasticity may also negatively impact on pain and spasticity (Brown and Weaver, 2012) when maladaptive synaptogenesis occurs. Changes in levels of neuromodulators including serotonin, dopamine, and noradrenaline are crucial facilitators of neuroplasticity (Azmitia, 1999; Nitsche et al., 2006; Marzo et al., 2009). Importantly, each of these neuromodulators engages in diverse physiological functions, including cognition, mood regulation, movement, and somatosensation; the therapeutic potential in targeting these neuromodulatory systems is hampered by their diverse physiological roles.

Different diet regimes hold promise as non-pharmacological alternatives to treat neural injury. Metabolism influences brain activity (Ruskin and Masino, 2012), and metabolic functions, in turn, depend on the diet (Flint et al., 2015). Studies investigating the clinical utility of metabolic therapies, such as the ketogenic diet (KD), indicate effectiveness in epilepsy, brain cancer, type 2 diabetes, and neurodegeneration (Barañano and Hartman, 2008). KD promotes the use of ketone bodies as energy sources by minimizing glucose metabolism and increasing ketolysis. By replacing glucose with ketone bodies, a direct consequence is enhanced cellular capacity in energy generation, increasing the availability of high-energy molecules including ATP and phosphocreatine (Ruskin and Masino, 2012). Other work has shown an indirect link between KD and the increase in inhibitory neuromodulators such as adenosine and GABA (Yudkoff et al., 2007). Hence, KD may be associated with a role in balancing neuronal excitability. In SCI, KD increased mobility, range of motion, and dexterity in the forepaws of rats following a high cervical SCI (Streijger et al., 2013). Interestingly, a combinatorial therapy that included KD, ibuprofen, ghrelin, and a peptide (C16), did not improve motor function (Streijger et al., 2014). However, there has been little research on the effects of KD on lumbar SCI or peripheral nerve injury. This is important since cervical segments control precision movements of the digits, during grasp for example, compared with more rhythmic movements of the hindlimbs that can be generated by spinal circuits.

The current study explores the therapeutic potential of KD in regulating motor and pain dysfunctions in nerve injuries. In C57BL/6 mice, two of the most common nerve injury models including a thoracic hemisection (SCI) and the spared nerve injury (SNI) were performed to mimic damage to the CNS and peripheral nervous system, respectively. A battery of sensory and motor behavioral tests were performed before and after the injuries to test baseline and postinjury effects of KD. The levels of neuromodulators were compared between mice fed standard chow and KD.

## Materials and Methods

All animals used were C57/BL6 male mice between 8 and 16 weeks of age during testing. All animal experiments were approved by the University of Calgary animal care committee and are catalogued under the protocol AC15-0026.

### Ketogenic diet administration

The following two standard diets were used with the animals in this study and were given *ad libitum*: Pellet diet (PD) and 6:1 KD. All animals were initially fed a standard pellet diet *ad libitum* [Pico-Vac Mouse Diet 20 (Lot 5062), LabDiet] given in the hanging feeding rack of the cages. KD animals were fasted for 1 evening (12 h), a week before testing and were given ∼15 g of frozen KD chow daily (catalog #S3666, Bio-Serv) to ensure ketone blood levels were above the threshold for ketosis (Smith et al., 2016). Nutritional composition and macronutrient levels are listed in Table 1.

**Table 1:** Nutritional composition of administered diets

Macronutrients	PD (LabDietmousediet 20)	KD (Bio-ServF3666)	Measurement
Protein	24.65%	8.60%	%
Fat	13.21%	75.10%	%
Carbohydrate	62.14%	3.20%	%
Other			
Fiber	4.00%	4.80%	%
Ash	6.10%	3.00%	%
Moisture	12.00%	<10%	%
Amino acids			
Alanine	1.15%	0.23%	%
Arginine	1.22%	0.31%	%
Aspartic Acid	2.19%	0.55%	%
Cystine	0.28%	0.03%	%
Glutamic acid	4.34%	1.73%	%
Glycine	0.96%	0.21%	%
Histidine	0.5%	0.23%	%
Isoleucine	0.97%	0.47%	%
Leucine	1.56%	0.71%	%
Lysine	1.16%	0.63%	%
Methionine	0.7%	0.22%	%
Phenylalanine	0.9%	0.38%	%
Proline	1.47%	0.87%	%
Serine	1.03%	0.48%	%
Threonine	0.77%	0.37%	%
Tryptophan	0.26%	0.1%	%
Tyrosine	0.59%	0.48%	%
Valine	1%	0.55%	%
Minerals			
Calcium	0.81%	0.57%	%
Chloride	0.42%	0.17%	%
Copper	13 ppm	6.6 ppm	ppm
Chromium	0.81 ppm	2.2 ppm	ppm
Fluoride/fluorine	10 ppm	0.0 ppm	ppm
Iodine	1.5 ppm	0.2 ppm	ppm
Iron	220 ppm	38.7 ppm	ppm
Magnesium	0.16%	0.56%	%
Manganese	85 ppm	63.6 ppm	ppm
Phosphorus	0.6%	0.49%	%
Potassium	0.7%	0.39%	%
Selenium	0.3 ppm	0.19 ppm	ppm
Sodium	3000 ppm	1128 ppm	ppm
Sulfur	3400 ppm	366 ppm	ppm
Zinc	87 ppm	36 ppm	ppm

Comparison table of the two diets used throughout this study. All values have been converted to the percentage based on weight from company data sheets of the diets. PD Pico-Vac Mouse Diet 20 from LabDiet compared with KD #F3666 6:1 from Bio-Serv. Diets have been sorted into four categories for readability: Macronutrients, Other, Amino acids, Minerals.

### Ketone blood sampling

Blood ketone concentrations were sampled using blood draws from the tail. Approximately 1.5–2 µl was extracted by making a superficial cut ∼1 mm from the end of the tail using a #10 scalpel blade (catalog #10 010–00, Fine Science Tools). The blood was massaged out of the tail, and this was tested using a ketone blood monitor (Freestyle Precision Neo, Abbott Laboratories) and keto testing strips (Abbott blood β-ketone test strip, Abbott Laboratories). All values were recorded in the software for offline analysis.

### HPLC tissue collection

In a cohort of 26 animals, mice were randomized and coded before being sacrificed after 28 d of being in ketosis. KD animals underwent the standard KD diet paradigm as outlined above. On the day of tissue collection, animals were sacrificed using high doses of isoflurane (5%) in an induction chamber. The spinal column was extracted by cutting the sacrum and cervical vertebral levels using large scissors. The spinal cord was extracted using a 10 ml syringe loaded with aCSF with fluid pressure initiated in the lumbar region. The spinal cord was trimmed to contain only the lumbar enlargement (L1–L6), and the spinal cords were flash frozen in liquid nitrogen. The spinal cord was analyzed for biogenic amines by modifications of a previously reported method (Parent et al., 2001). Tissue was homogenized in ice-cold 0.1N perchloric acid containing EDTA (10 mg%) and ascorbic acid (50 μm). The homogenate was centrifuged and 10 µl of supernatant was used in the HPLC assay using an Atlantis dC18 column (Waters) and an electrochemical detector.

### Surgical intervention

All surgical procedures were performed using aseptic techniques under isoflurane anesthesia between 1% and 2% delivered by 0.4 L/min medical grade oxygen (100% oxygen; Vitalair 1072, Hosokawa). The area of surgical intervention was shaved using animal shears, cleaned with betadine 5% solution, and sterilized with 95% ethanol.

### Spared nerve injury

Once anesthetized and prepared, a superficial cut of 1 cm was made in the left hindleg in the skin above the midline of the femur, above the knee joint to below the hip joint. The skin was resected from the fascia by blunt dissection to properly view the musculature of the triceps surae and the biceps femoris. The muscles were separated along where both intersect. The sciatic nerve was isolated between the trifurcation of the common peroneal (CP)/Tibial nerve (Tib) and the sural nerve with surrounding fascia removed. Both the CP and Tib were ligated using one 6–0 suture (catalog #18 020–60, Fine Science Tools). The nerves were then cut on the distal end of the suture to create a full transection of both the Tib and CP while leaving the sural nerve intact. For sham procedures, the nerves were exposed and identified but were left intact. Open wounds and musculature were sutured using a 4–0 dissolvable suture in an interrupted fashion with tissue adhesive (Vetbond, 3M) on the skin and sutures to secure the knots. SNI procedures were verified during surgeries and *post hoc*.

### Spinal cord injury

A superficial incision was made 2 cm in length along the midline of the thoracolumbar vertebral column (T10–T13) where the curvature of the back is most pronounced. The skin and fascia were dissected away from the underlying musculature to expose the underlying vertebrae and surrounding muscles. An incision along both sides of the midline of the T10–T11 dorsal spinous processes was performed, and a muscular resection exposed the underlying vertebrae. The underlying vertebrae were blunt dissected using curved forceps (catalog #11 154–10, Fine Science Tools) to expose the dorsal processes and the intervertebral disk. The intervertebral disk was removed, and the underlying spinal cord was exposed. Partial laminectomies (half of the vertebrae) were performed between the T10–T11 vertebrae to expose sufficient spinal cord for a hemisection while maintaining structural integrity and preserving the dorsal roots (rongeur, catalog #16 221–14, Fine Science Tools,). The spinal cord hemisection was performed using a 30 gauge one-half inch needle (catalog #305106, BD) inserted into the midline of the spinal cord until the ventral vertebrae were reached. The cord was then cut in a perpendicular motion toward the lateral edge of the vertebrae, hemisecting the spinal cord on the left side of the animal. For sham procedures, the spinal cord was exposed with a laminectomy, but no hemisection was performed. Animals were given buprenorphine at a dose of 0.1 mg/kg, i.p., before removing from isoflurane anesthetic. Animals were assessed daily after injuries to verify proper pain management and whether any postoperative complications had occurred. No extra analgesic was required following the day of surgery. SCIs were verified visually at the time of surgery and *post hoc*.

### Behavioral and pain testing

#### Randomization

All experiments were randomized and blinded. Randomization was performed using a random number generator (www.random.org) each day of experimentation to make sure animals were not habituated to specific boxes or positions in the tests. Animals were tested in cohorts of 12, divided into the following three groups: SHAM, Injury+PD, and Injury+KD. All animals in each cohort were assigned a reference number between 1 and 12. On testing days, the random number generator provided a 2 × 6 matrix of randomly assigned numbers between 1 and 12 indicative of either the position of placement in the testing apparatus or the order in which an animal was tested. This method ensured that animals were not habituated to either the specific location in the testing apparatuses or the time of day that the testing was performed.

#### von Frey

Mechanical allodynia was assessed by measuring the hind paw withdrawal threshold to von Frey hairs (vFhs). Animals were placed individually into Plexiglas testing chambers on a raised mesh surface to allow access to the hindpaws and were habituated for 30 min before testing. Baseline mechanical sensitivity was determined for each animal before the injuries. A range of vFhs were used (0.2–2 g), the presence or absence of a withdrawal response was recorded, and the withdrawal threshold was calculated using the up-down method of Dixon (Chaplan et al., 1994).

#### Open field test

For open field tests (OFTs), animals were habituated to the room for 1 h before tests were administered. Animals were placed into the open field boxes (Cleversys Systems) four boxes at a time. The open field protocol was set for 30 min from the time of insertion into the arena. The center of the box was defined by 25% of the size of the total arena in the center of the box. The periphery was defined as the total area of the box minus the center area and covers the periphery around the center to the walls of the open field.

#### Ladder rung

For ladder-rung testing and scoring, we adapted previously reported methods (Metz and Whishaw, 2009). The ladder-rung system was constructed of Plexiglas sides and used a 2-mm-diameter round, 100-mm-long steel bar for the rungs. The rungs were spaced randomly, and the spacing was not changed throughout the experiments. Animals were habituated and exposed to the test on 3 separate testing days before injuries occurred. During habituation and data acquisition, animals were passed over the ladder rung a total of three times, and the average step scores and faults were determined daily. Animals were introduced into the right side of the apparatus to allow full visualization of the injured left hindlimb. The order of the animals placed on the ladder rung was randomized on each testing day. Each session was recorded using a high-speed camera (VIXIA HF R52 HD, Canon) recorded at 60 frames/s with a 1080p resolution. Scoring was performed *post hoc* by separate personnel who were blinded to the condition of the mice. Scoring was based on the 6 point scale outlined in the study by Metz and Whishaw (2009; Table 2), with a score of 0 denoting when a hindlimb completely missed the rung leading to a break in the weight-bearing gait. By comparison, a score of 5 would indicate a slight malposition of the hindlimb on a rung of the test, with a score of 6 being perfect placement on the ladder rung. Decoding and interpretation of the data were performed by other personnel in the laboratory to minimize bias.

**Table 2:** Ladder-run foot scoring scale

Score	Metz scale2009	Explanation
0	Total miss	0 points were assigned when the limb completely missed a rung (i.e. it did not touch it), and a fall occurred. Afall was defined as a limb deeply falling in between rungs with body posture and balance being disturbed.
1	Deep slip	The limb was initially placed on a rung, then slipped off as the mouse began to bear weight, causing theanimal to fall.
2	Slight slip	The limb was placed on a rung, then slipped off as the mouse began to bear weight, but it did not resultin a fall or interrupt the step cycle. In this case, the animal was able to maintain balance and continuea coordinated gait.
3	Replacement	The limb was placed on a rung, but before it was bearing weight it was quickly lifted and placed onanother rung.
4	Correction	The limb was aimed for one rung but was then placed on another rung without touching the first one.Alternatively, a score of 4 was recorded if a limb was placed on a rung and was quickly repositionedwhile remaining on the same rung.
5	Partialplacement	The limb was placed on a rung with either wrist or digits of the forelimb or heel or toes of the hindlimb.
6	Correctplacement	The midplantar surface of a limb was placed on the rung with full weight support.

This 6 point scale was used to score the mouse hindlimb positioning when crossing over the ladder-run test, 0 being the complete miss of the hindpaw placement on the ladder rung, up to a maximum score of six with correct paw placement achieved. This scale was used throughout the experiments and was adapted from Metz and Whishaw (2009).

### Statistics

All data were tested for normality using the D’Agostino and Pearson tests. Data that were normally distributed were subsequently analyzed using parametric tests; nonparametric tests were used to analyze data that were not normally distributed. Analysis of behavioral data was performed using repeated-measures two-way ANOVAs with Bonferroni multiple comparisons or a mixed-effects analysis. The factors were treatment (KD vs PD) and time (before and after injuries). Analysis of baseline behavioral changes, neuromodulator levels (HPLC), and ketone blood concentrations between KD and PD animals were performed with unpaired *t* tests or Mann–Whitney tests. For all statistical analyses, Prism 8 software (GraphPad Software) was used. A significance level of *p* < 0.05 was used throughout represented by an asterisk in figures.

## Results

### Mice fed ketogenic diet had increased ketone levels and no changes in body weight

**Figure 1.**
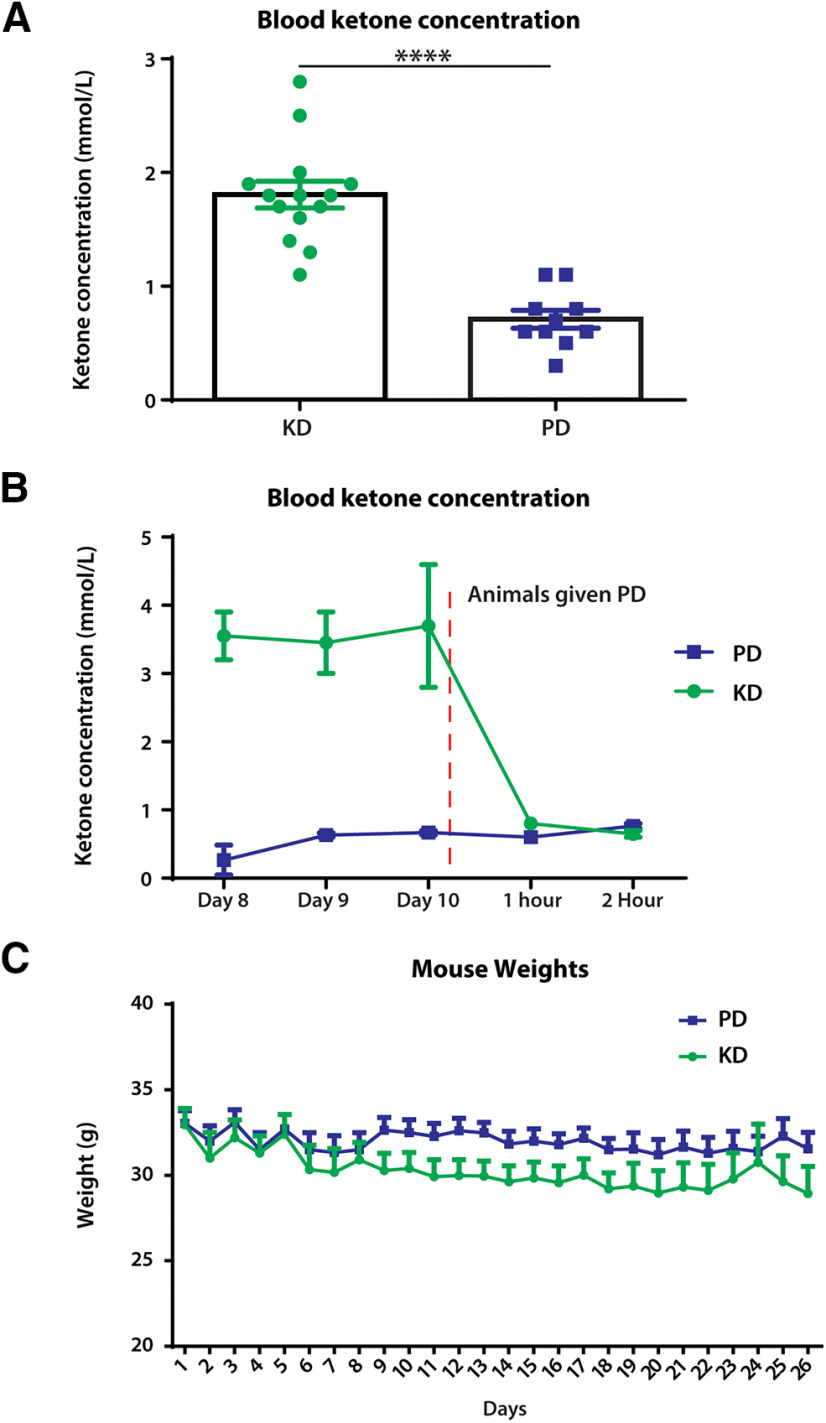
Blood ketone levels and stability 1 week after administration of ketogenic diet in uninjured mice. ***A***, Blood samples were collected from the tail vein and determined by ketone testing strips and monitoring. Significantly higher ketone blood concentrations were found in mice fed the ketogenic diet compared with a conventional pellet diet. *N* = 10 each for KD and PD; ****p < 0.0001. ***B***, Mice fed a ketogenic diet reliably remained in ketosis with KD being introduced 7 d prior. In addition, within 1 h of being given a pellet diet, mice were no longer in a state of ketosis. *N* = 2 for KD; *N* = 3 for PD. ***C***, Comparison of body weights between mice who were fed the ketogenic and pellet diets without surgical intervention; no significant difference was detected at each time point between the diets. *N* = 6–10 each for PD and KD. Error bars indicate the mean ± SEM.

We first examined the efficacy of the KD in producing a steady ketosis state in uninjured mice. All animals were tested for blood ketone levels before and following diet administration (Fig. 1*A*). Animals fed a KD showed the concentration of blood ketones (unpaired *t* test: *t* = 6.624, df = 18, *p* < 0.0001), reaching an average of 1.8 mmol/L ketones that is indicative of ketosis (Ruskin et al., 2009). Animals fed a PD had an average blood ketone level of 0.7 mmol/L. When animals in ketosis were fed PD, they showed a decrease in blood ketone levels 60 min following ingestion (two-way repeated-measures ANOVA; time × diet: *F*_(4,12)_ = 28.51, *p* < 0.0001; time: *F*_(1.327,3.980)_ = 22.78, *p* = 0.0079; diet: *F*_(1,3)_ = 51.35, *p* = 0.0056; Fig. 1*B*), suggesting that maintenance of high blood ketone levels reliably indicates the state of ketosis. No significant difference in weight between animals fed either KD or PD was detected during the course of the study (mixed-effects analysis: time × diet, *p* = 0.0164; time, *p* = 0.005; diet, *p* = 0.131; Fig. 1*C*).

### Levels of monoamines in the mouse spinal cord were unaffected by the ketogenic diet

To investigate whether changes in diet influence expression of monoamines, crucial neuromodulators of locomotion and nociception, we next measured levels of monoamines within the lumbar spinal cord of mice fed with KD or PD using HPLC.

**Figure 2.**
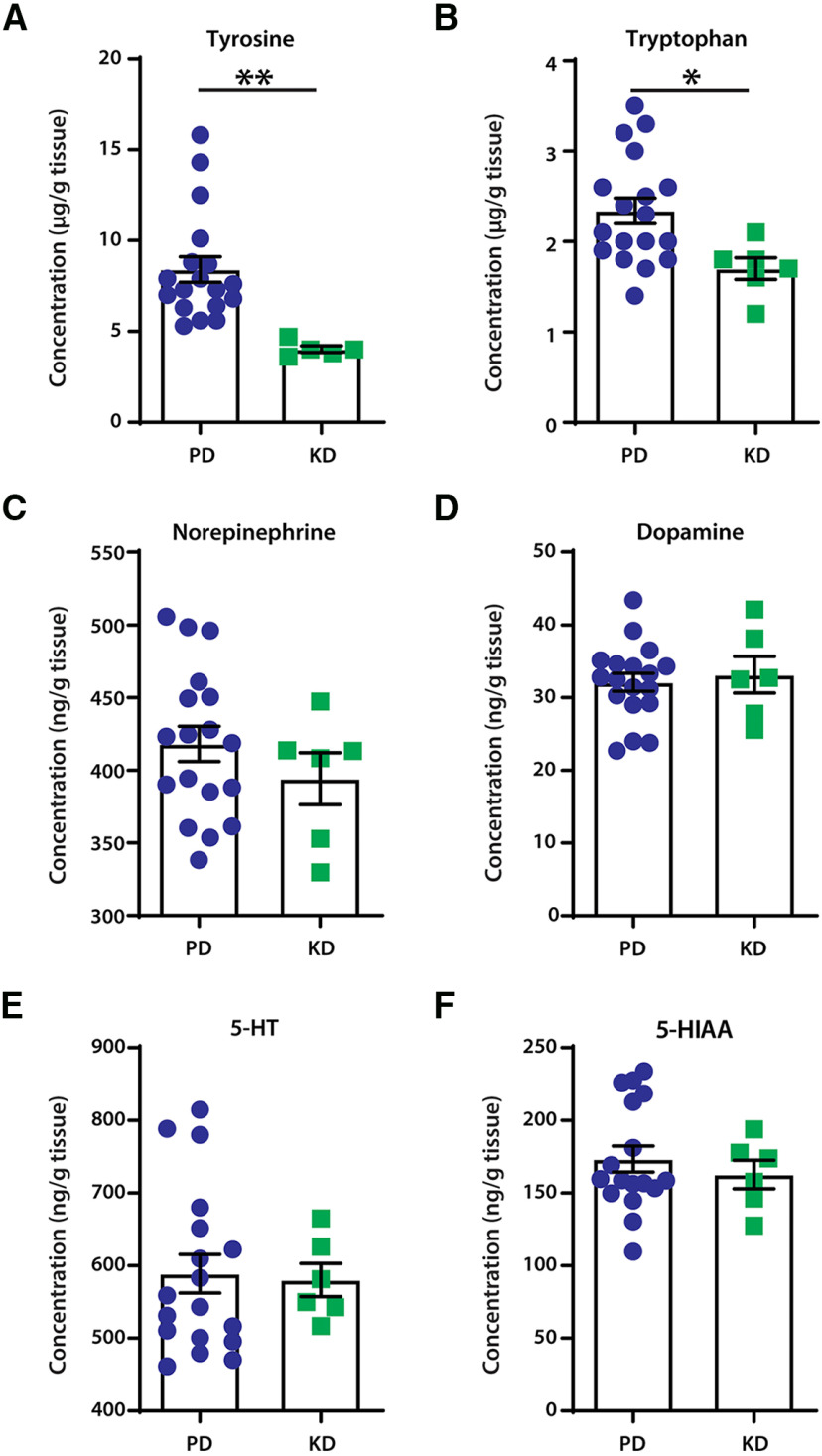
The effect of diets on neuromodulator content in the lumbar spinal cord of mice. ***A***, ***B***, Mice fed the ketogenic diet had significantly lower content of the monoamine precursors tyrosine (***A***) and tryptophan (***B***); **p = 0.0041 and *p = 0.0209, respectively. ***C***–***E***, No changes were detected in the levels of monoamines noradrenaline (***C***), dopamine (***D***), and serotonin (5-HT; ***E***). There were also no changes in the content of the main metabolite of serotonin, 5-hydroxyindoleacetic acid (5-HIAA; ***F***). *N* = 18 for PD; *N* = 6 for KD. Error bars indicate the mean ± SEM.

Mice fed KD had significantly lower levels of the essential amino acids tyrosine (unpaired *t* tests: *t* = 3.221, df = 21, *p* = 0.0041; Fig. 2*A*) and tryptophan (unpaired *t* tests: *t* = 2.489, df = 22, *p* = 0.0209; Fig. 2*B*). This suggests a lower availability of substrates for the production of monoamines. However, no changes were detected between the diet groups in levels of noradrenaline (unpaired *t* test: *t* = 1.022, df = 22, *p* = 0.3177; Fig. 2*C*), dopamine (unpaired *t* test: *t* = 0.4043, df = 22, *p* = 0.6899; Fig. 2*D*), or serotonin (unpaired *t* test: *t* = 0.1737, df = 22, *p* = 0.8637; Fig. 2*E*). Furthermore, levels of the main metabolite of serotonin, 5-hydroxyindoleacetic acid, remained unaltered (unpaired *t* test; *t* = 0.6498, df = 21, *p* = 0.5228; Fig. 2*F*).

### The ketogenic diet had no effect on sensorimotor recovery in the context of a peripheral nerve injury

We sought to understand the effects of KD in sensorimotor recovery following peripheral nerve injury. SNI was performed as animals exhibit motor deficits and pronociceptive phenotypes.

**Figure 3.**
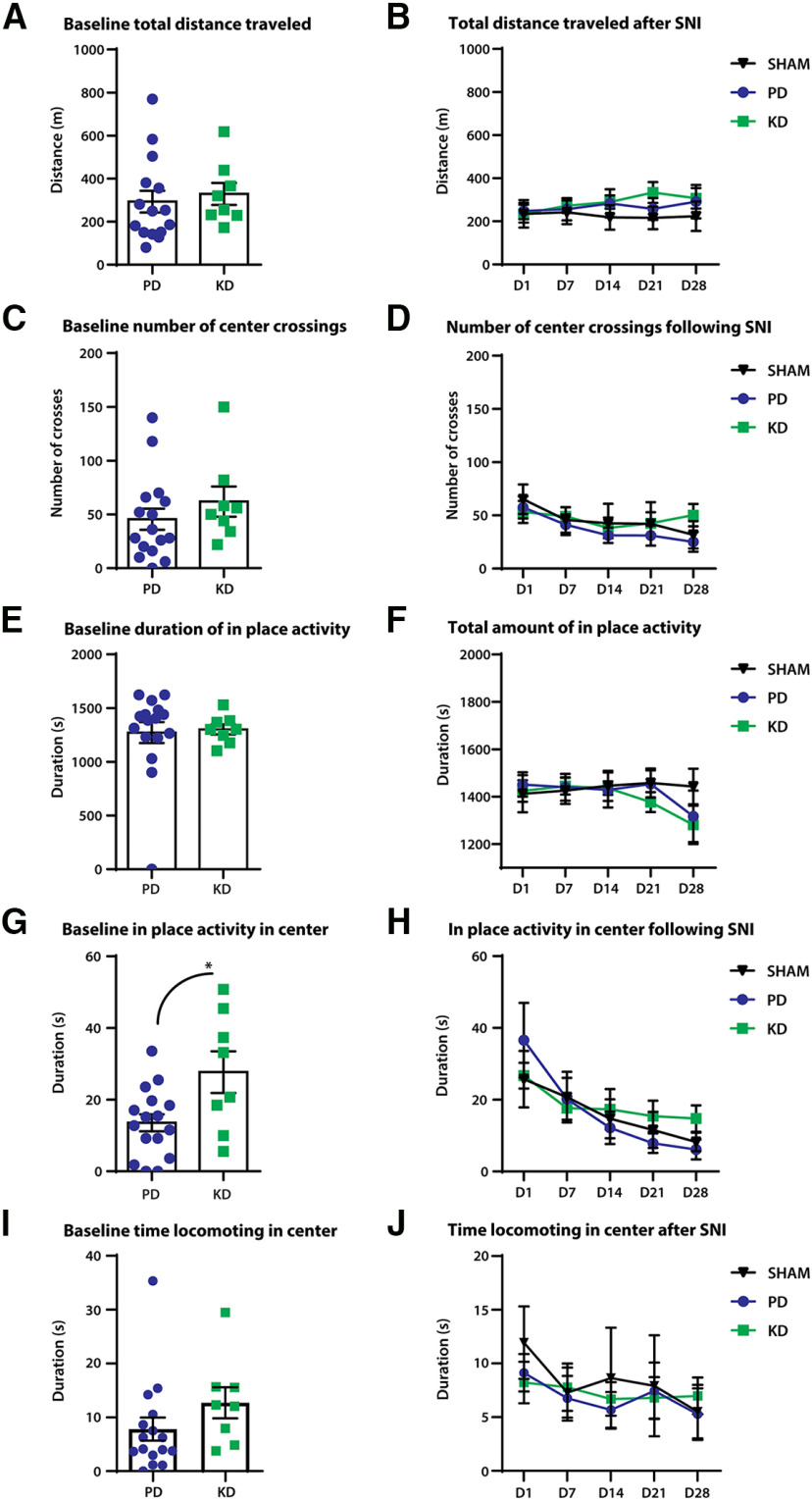
The effect of diet on open-field locomotor behaviors in a model of peripheral nerve injury. Peripheral nerve injury was modeled by SNI; comparison of general locomotor behaviors were made at before (baseline) and after SNI. ***A–F***, No significant differences were observed between the diets in distance traveled (***A***, ***B***), duration of in-place activity (***C***, ***D***), and number of crossings from the periphery of the open field box to the center (***E***, ***F***). ***G***, At baseline, mice fed the ketogenic diet displayed more in-place activity in the center of the box. *p = 0.0138. ***H***, However, no effects of diet were observed after SNI between mice fed a pellet or ketogenic diet. Duration of locomotion in the center of the open field box is a proxy measure of anxiety-like behaviors. The more time spent in the center of the box suggests less anxiety as mice innately avoid bright open spaces. ***I***, At baseline, no significant movement to the center was observed; *p* = 0.054. ***J***, No effects of the diet were observed after SNI. *N* = 7–8 for SHAM; *N* = 8 for SNI + PD; *N* = 8 for SNI + KD. Error bars indicate the mean ± SEM.

We first examined general locomotion before and after SNI surgeries using open field tests. Mice were allowed to move freely in the open field boxes, and activity was recorded for 30 min. Before SNI surgeries (baseline), mice showed no difference in total distance traveled (Fig. 3*A*), crossing from the periphery of the box to the center (Fig. 3*C*), and duration of in-place activity in the OFT (Fig. 3*E*). Interestingly, the duration of in-place activity in the center, when the animal is active, but not moving outside of a prescribed area, was increased in KD mice compared with PD mice (unpaired *t* test: *t* = 2.676, df = 22, *p* = 0.0138; Fig. 3*G*). No significant time spent in the center locomoting was observed (Mann–Whitney test, *p* = 0.054; Fig. 3*I*). These results indicate that at baseline, mice fed a KD may be less opposed to spending time in the center of the box, which is suggestive of an anxiolytic effect.

General locomotive behaviors were tracked for 28 d after SNI surgeries. No differences were detected between the diets in total distance traveled (Fig. 3*B*), duration of in-place activity (Fig. 3*D*), and crossing over from the periphery to the center (Fig. 3*F*). No changes in the duration of in-place activity (Fig. 3*H*) or locomotion (Fig. 3*J*) in the center were observed.

**Figure 4.**
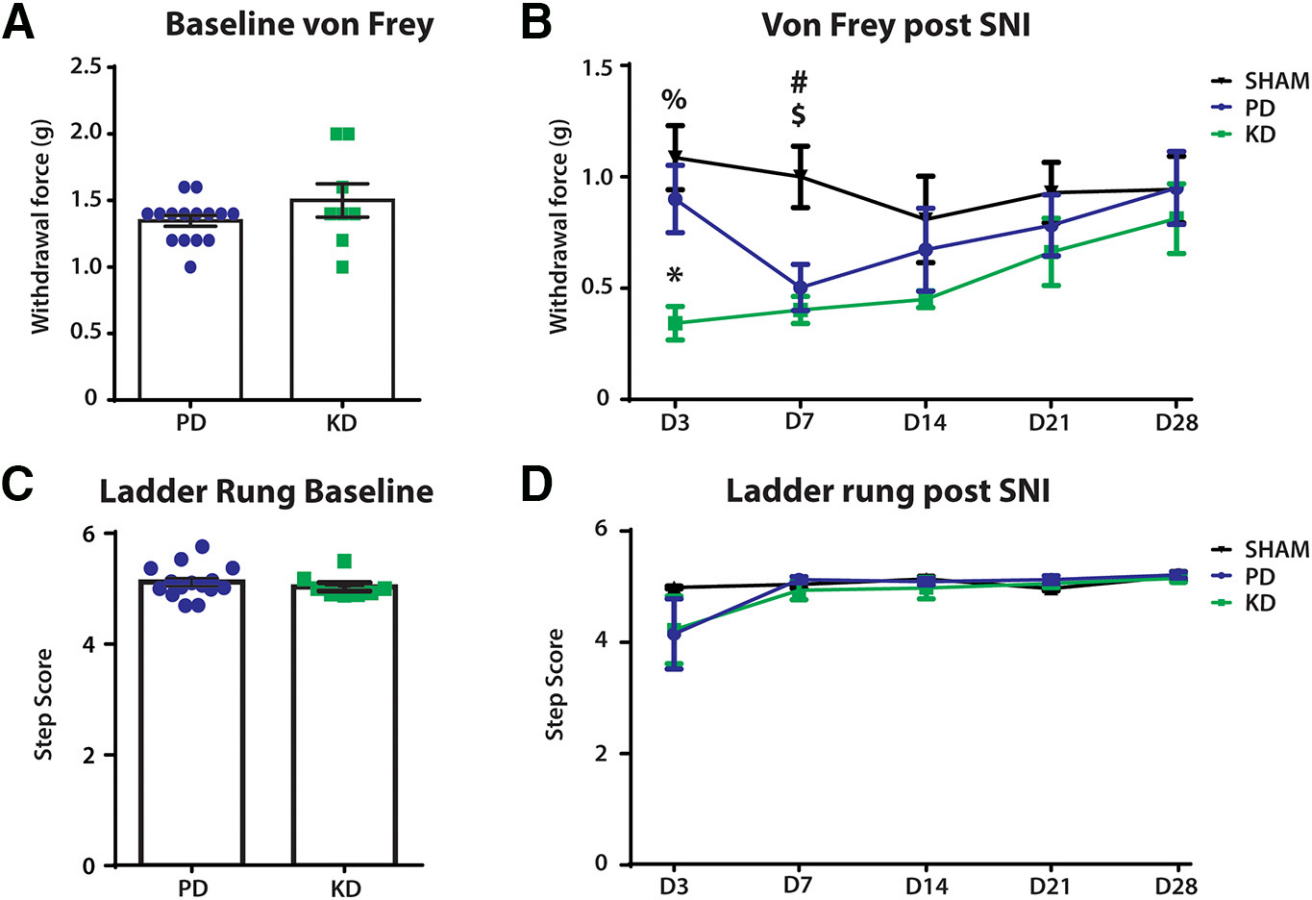
The effect of diet on other sensorimotor behaviors before and after peripheral nerve injury. Comparison of mechanical withdrawal threshold (von Frey hair test) and fine motor behaviors (ladder rung) made before (baseline) and after SNI. ***A***, No difference in the mechanical withdrawal threshold between the diets at baseline. ***B***, Mechanical withdrawal threshold was significantly lower in SNI mice compared with sham mice, indicating the development of mechanical allodynia (pain evoked by normally innocuous stimulations). *Post hoc* Bonferroni analysis revealed a significant difference between the SHAM+PD and SNI+KD groups on days 3 and 7 after SNI; %*p* = 0.0038, #*p* = 0.0117, respectively. The SNI+PD group also had a lower mechanical withdrawal threshold compared with SHAM+PD on day 7 after SNI, $*p* = 0.0428. Three days after SNI, the SNI+KD group also had significantly lower mechanical withdrawal threshold compared with the SNI+PD group; *p = 0.0231. ***C***, During Ladder rung, there was no difference in the step score of animals at baseline between animals fed the KD vs PD. ***D***, Over the course of 28 days recovery, diet produced no significant changes in recovery. *N* = 7 for SHAM group, *N* = 8 for SNI+KD and SNI+PD groups. Error bars indicate the mean ± SEM.

To investigate the effect of diet on nociception, von Frey hair tests were performed to measure mechanical withdrawal thresholds at baseline and after SNI surgeries. There were no differences at baseline (Fig. 4*A*), but the mechanical withdrawal threshold was significantly lower after SNI (two-way repeated-measures ANOVA: time × treatment: *F*_(8,80)_ = 1.343, *p* = 0.2347; time: *F*_(2.98,59.6)_ = 2.29, *p* = 0.0879; treatment: *F*_(2,20)_ = 7.185, *p* = 0.0045; Fig. 4*B*). *Post hoc* analysis revealed that mice fed a KD had significantly lower mechanical withdrawal thresholds 3 d after SNI compared with a PD (KD vs PD, 0.9 vs 0.5575 g; *p* = 0.0231), but not in the latter time points. This indicates an initial worsening of allodynia (pain evoked by normally non-noxious stimulations) after SNI in KD animals.

To quantify the effects of diet on the recovery of fine motor skills, the ladder-rung test was used to qualitatively score the motor ability of the animals. Animals showed no statistical difference before SNI between groups fed either a PD or KD at baseline (unpaired *t* test: *t* = 0.7329, df = 21, *p* = 0.4717; Fig. 4*C*). Following injury, animals initially had a reduced ability to cross the ladder-run platform, which recovered over time. However, no difference in recovery was observed between animals fed KD or PD (Fig. 4*D*; two-way ANOVA; time × treatment: *F*_(6,60)_ = 1.796, *p* = 0.1152; treatment: *F*_(2,20)_ = 1.882, *p* = 0.1783).

Before the commencement of sensorimotor behavior tests, animals were in ketosis with ketone levels of 2.0 mmol/L ± 0.580 compared with the PD-fed animals with mean ± SD ketone levels of 0.653 mmol/L ± 0.195 mmol/L (unpaired *t* test: *t* = 9.228, df = 22, *p* < 0.0001). Before sacrificing the animals, we took a blood sample and determined the ketone concentrations. The PD average was 0.725 mmol/L ± 0.084, while the KD blood ketone concentration was 2.238 mmol/L ± 1.509 (unpaired *t* test: *t* = 3.954, df = 21, *p* < 0.0007).

### The ketogenic diet had no effect on sensorimotor recovery in the context of a spinal cord injury

**Figure 5.**
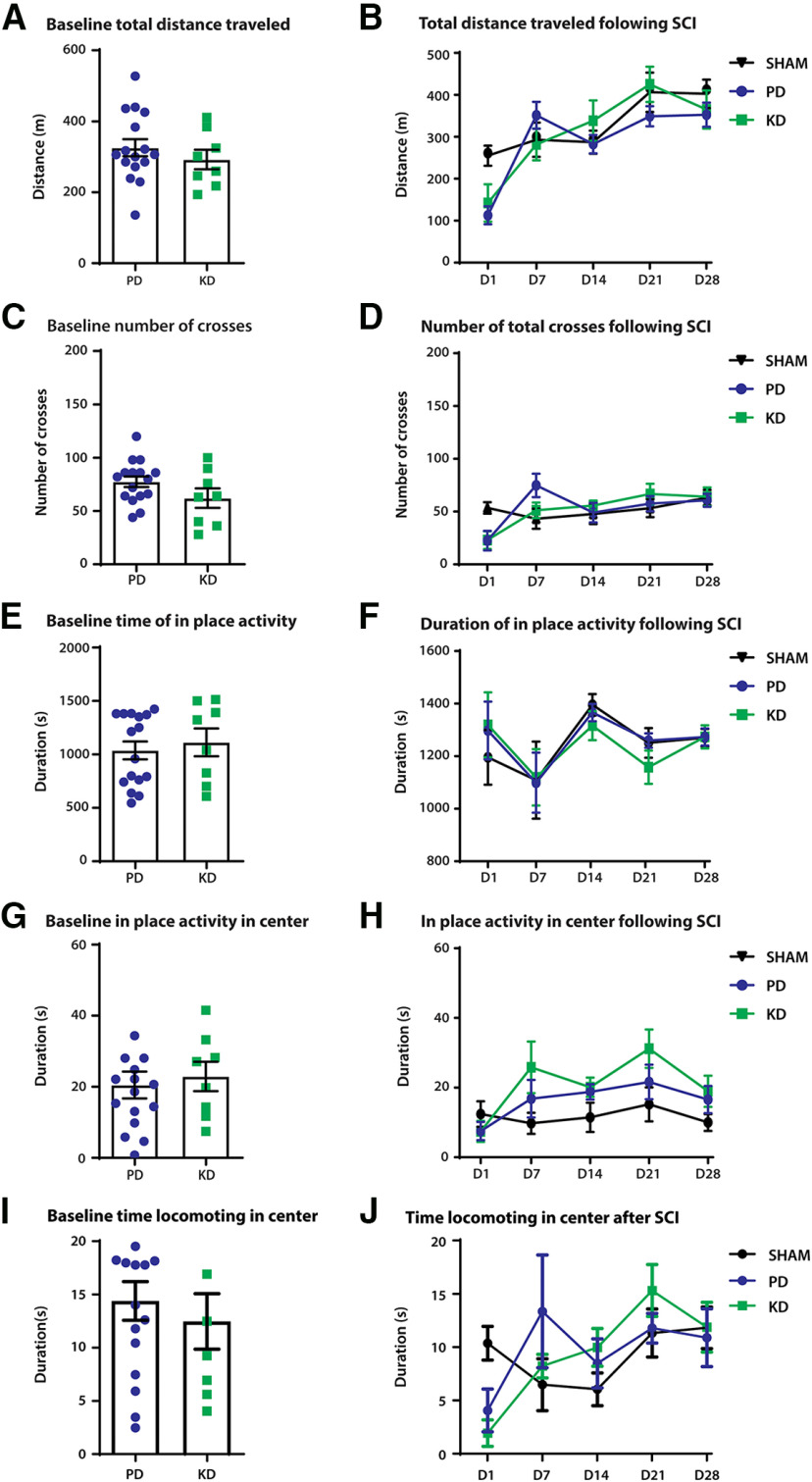
The effect of diet on open-field locomotor behaviors in a model of spinal cord injury. SCI was modeled by a hemisection at T12–T13, on the left side of the spinal cord. A comparison of general locomotor behaviors was made before (baseline) and after SCI. ***A***, ***B***, Distance traveled after 30 min in the OFT; there was no significant difference at baseline between PD-fed and KD-fed animals (***A***) or following injury (***B***). ***C***, ***D***, No difference in the number of crosses from the periphery of the open field box to the center. ***E***, ***G***, At baseline, mice fed the ketogenic diet displayed no differences in activity in place in the entirety of the arena (***E***) or in the center of the box (***G***). ***F***, ***H***, No significant effects of diet were observed for in-place activity after SCI between PD- and KD-fed mice in either the arena (***F***) or the center of the box (***H***). The duration of locomotion in the center of the open field box is a proxy measure of anxiety-like behaviors. The more time spent in the center of the box indicates less anxiety as mice innately avoid bright open spaces. ***I***, Nonsignificant difference at baseline in mice fed a KD versus mice fed a PD during locomotion in the center of the open field (Student’s *t* test, *p* = 0.548). ***J***, No effects of the diet were observed after SCI except for in the time domain; two-way ANOVA, *p* = 0.008. There were no significant differences between animals in the KD group and the PD group following injury (two-way ANOVA, *p* = 0.8994). *N* = 8 for SHAM, *N* = 8 for the SCI+PD group, *N* = 8 for SCI + KD group. Error bars indicate the mean ± SEM.

We next sought to examine the effects of a KD on neurotrauma to the CNS, in particular the thoracolumbar spinal cord. We used the spinal cord hemisection model to produce a repeatable injury. We first examined the motor behavior of the mice in open field boxes. Before SCI (baseline), mice showed no significant differences in motor behaviors when fed KD or PD. Specifically, we found no difference in the distance in the open field (Fig. 5*A*), time spent in place in the open field (Fig. 5*C*), total crosses of the outside periphery to the inside area (Fig. 5*E*), the duration of in-place activity in the center of the open field (Fig. 5*G*), and time spent locomoting in the center of the box (Fig. 5*I*).

General locomotor behaviors were tracked for 28 d following SCI. No differences were detected between the diets in total distance traveled in the open field (two-way ANOVA; time × treatment: *F*_(8,84)_ = 2.617, *p* = 0.0131; treatment: *F*_(2,21)_ = 0.5886, *p* = 0.5640; Fig. 5*B*), duration of in-place activity (Fig. 5*D*), crossing from the periphery to the center of the box (two-way ANOVA; time × treatment: *F*_(8,84)_ = 2.907, *p* = 0.0065; treatment: *F*_(2,21)_ = 0.007431, *p* = 0.9926; Fig. 5*F*). No differences were present between treatment groups in the duration of in-place activity in the center of the box (Fig. 5*H*), along with no difference in the time spent locomoting in the center of the open field (Fig. 5*J*).

**Figure 6.**
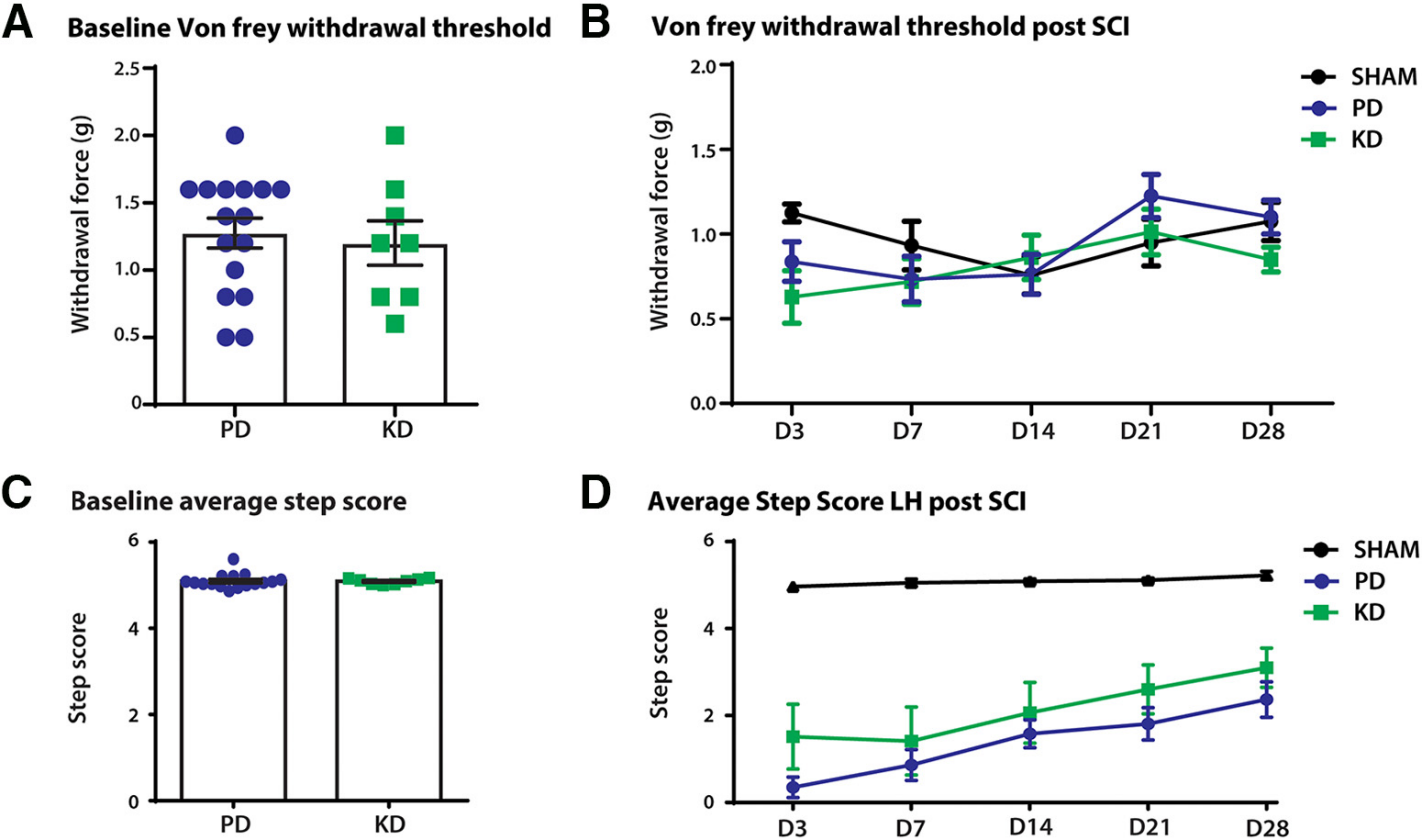
The effect of diet on other sensorimotor behaviors before and after spinal cord injury. SCI was modeled by a hemisection T12–T13, left side of the spinal cord. Comparison of mechanical withdrawal threshold (von Frey hair test) and fine motor behaviors (ladder rung) was made at before (baseline) and after SNI. ***A***, ***B***, No difference in mechanical withdrawal threshold between the diets at baseline (***A***) or after SCI (***B***). ***C***, There was no difference in the step score of animals at baseline between animals fed the KD versus those fed the PD. ***D***, Diet produced no significant changes over the course of 28 d on the rehabilitation of motor recovery. *N* = 8 each for SHAM, SNI+KD, and SNI+PD. Error bars indicate the mean ± SEM.

vFh tests were performed to investigate nociception following SCI. At baseline, animals fed PD chow did not show any marked differences from animals fed a KD (unpaired *t* test: *t* = 0.385, df = 22, *p* = 0.7038, Fig. 6*A*). Following SCI, no significant changes to the withdrawal threshold over the course of recovery were found (two-way ANOVA, time × treatment: *F*_(6,84)_ = 0.9613, *p* = 0.457; trea.tment: *F*_(1,14)_ = 2.02, *p* = 0.178; Fig. 6*B*).

To quantify the effects of diet on the recovery of fine motor skills, the ladder-run test was used to qualitatively score the motor ability of the animals. Animals showed no statistical difference between groups fed either PD or KD at baseline (unpaired *t* test: *t* = 0.1162, df = 22, *p* = 0.9086, Fig. 6*C*). Following injury, both injury groups had a reduced ladder-run score, compared with the sham mice (two-way ANOVA; time × treatment: *F*_(8,84)_ = 5.024, *p* < 0.0001; treatment: *F*_(2,21)_ = 23.87, *p* < 0.0001, Fig. 6*D*). However, no difference in recovery was observed between animals fed a PD or KD in SCI animals.

Preinjury, animals were in ketosis with mean ± SD ketone levels of 1.97 ± 1.25 compared with the PD-fed animals with ketone levels of 0.75 ± 0.241 (unpaired *t* test: *t* = 3.651, df = 21, *p* < 0.0015). Before sacrificing the animals, we obtained a blood sample and determined the ketone concentrations. The PD average was 0.750 mmol/L ± 0.259, while the KD concentration was 2.063 mmol/L ± 0.830 (unpaired *t* test: *t* = 4.752, df = 16, *p* < 0.0002).

## Discussion

There is an urgent need to assess the potential of new therapeutic solutions, including diet-based interventions, to improve recovery following neurotrauma. Our study concluded there were minimal effects of the KD, a common form of metabolic therapy, on recovery from neural injuries (Streijger et al., 2013; Li et al., 2020). At baseline, before the injuries, we observed no significant differences in locomotor behaviors using the open field and ladder-run paradigms, or mechanosensory behaviors tested with von Frey hairs. Animals who were on the KD diet also had no differences in the levels of neuromodulators (dopamine, serotonin, and noradrenaline) in the spinal cord, although we did observe a decrease in levels of tyrosine and tryptophan. Similar reductions in tryptophan have been found in rats fed a KD (Heischmann et al., 2018). Following SNI-mimicking injury to the peripheral nervous system, an increase in mechanical allodynia (pain evoked by normally non-noxious stimulus) was observed in KD-fed animals compared with those fed a normal PD during the early phase of pain development. No other changes were detected in locomotor behaviors. In our SCI model, KD did not mitigate nociception or locomotor disability following thoracic spinal cord hemisection. Altogether, our data suggest that KD is not a suitable therapy for improving disability after peripheral or spinal cord injuries affecting the hindlimb.

This study was conceived on the premise that KD contributes to the reprogramming of cellular metabolism, thus modulating processes related to disorders of the CNS, including aberrant neuronal activity, neuroinflammation, and neurodegeneration (Cullingford, 2004). The KD replaces glucose with ketone bodies as the source for generating high-energy molecules, including ATP. It has been reported that by switching to ketone bodies, cellular energy production becomes more efficient (Ruskin and Masino, 2012). We hypothesized that this change in cellular metabolism would regulate cellular processes consequential to nerve injuries. Importantly, pain and motor deficits resulting from nerve injuries share similar cellular mechanisms. Immediately after a nerve is injured, neurodegeneration occurs followed by robust inflammatory processes, which aid the removal of cellular debris and subsequent sprouting of neural fibers (Benowitz and Popovich, 2011). These processes require precise temporal and spatial signaling of a plethora of trophic factors, relying on receptor binding on efferent neuronal targets for successful circuit remodeling (Hermann and Chopp, 2012). Importantly, errors in these intricate processes would result in aberrant neuronal function, thereby perpetuating motor deficits and pain following nerve injuries (Kohama et al., 2000; Hains et al., 2005). Often, these changes can occur quickly, for example leading to the hyperexcitability of neurons within 24 h of peripheral nerve injury (Iwata et al., 1999).

There are other reports which elucidated the molecular underpinnings in the benefits of KD. In a murine model of kainic acid-induced seizures, KD activates peroxisome proliferator-activated receptor-γ, which suppressed the expression of proinflammatory tumor necrosis factor-α and cyclooxygenase-2 (Jeong et al., 2011). Using the progressive genetic model of Alzheimer’s disease (APP/V7171 mutation), mice on the KD had overall lower levels of amyloid-β (Van der Auwera et al., 2005), a neurotoxin that on accumulation forms plaques and leads to cellular death (Mark et al., 1995). However, the same study also reported that KD did not alter behavioural outcomes including object recognition. Another study also found no improvements in memory functions in the APP/V7171 mutation model after a KD diet (Van der Auwera et al., 2005). An older study, using a different epileptic model (bicuculline-induced seizures) also found the effect of KD to be age dependent: KD initiated at postnatal day 16 protected animals against the tonic-extensor phase of bicuculline-induced seizures, whereas these effects were diminished if KD was initiated at postnatal day 26 (Uhlemann and Neims, 1972).

Our current study showed no overall improvements in nerve injury-induced sensorimotor deficits after KD, which is in contrast to other published observations. Rats fed a KD for 3–4 weeks showed reduced thermal hyperalgesia in a model of complete Freund’s adjuvant-induced chronic inflammation (Ruskin et al., 2009). In a murine model of obesity and prediabetes, the development of mechanical allodynia and loss of peripheral axonal termination was not observed in KD-fed animals, suggesting a neuroprotective mechanism (Cooper et al., 2018). Despite differences in an animal model of choice and testing modality, one experimental concern could be the duration of testing, as the pathogenesis of pain and motor deficits is dynamic and could differ between early and late phases of the injury (Kawasaki et al., 2008).

While no studies that we are aware of have addressed the effects of KD on thoracic SCI or peripheral nerve injury, there have been reports of the beneficial effects of KD on the recovery of reaching behavior in high cervical injuries in both mouse studies (Streijger et al., 2013) and human studies (Yarar-Fisher et al., 2018). These effects on reaching behavior in mice were confined to supination and grasping, suggesting regional effects on the cervical motor circuit. Other metrics showed a rapid increase in use behavior of the affected limb in KD cohorts. Importantly, our study concentrated on hindlimb, and connectivity onto the Central pattern generator (CPG) is different with large projections from the reticulospinal tract contributing to locomotor activity along with the inherent plasticity of the CPG itself (Girgis et al., 2007). Also, we used mice, whereas rats, which score lower on the BBB scale for motor recovery following SCI (Byrnes et al., 2010), were used in the forelimb study. To our knowledge, the time course of testing used in this study was comprehensive. The testing evaluated the most significant time points for motor recovery following injury, characterizing changes both before the onset of injury, and up to 4 weeks after. Most of the behavioral observations steadily plateaued before the end of the study. However, we do note that some of the metrics observed following SCI forelimb recovery occurred following 6 weeks of KD treatment. Surprisingly, none of these beneficial effects were observed if a KD was combined with other treatments (ibuprofen, ghrelin, and C16; Streijger et al., 2014). Future studies could extend the testing period beyond 4 weeks, and include measures of thermal sensitivity and neurite outgrowth to fully elucidate the effects of the KD. Our study used a hemisection model for SCI, producing a relatively mild injury and hindlimb-specific effects on ascending afferent projections. It is, therefore, possible that KD could be effective in a severe contusion model.

Another experimental consideration relates to the time required for animals to calibrate to the state of ketosis. Varying paradigms have been reported, ranging from introducing KD for 10 d to 4 weeks before the commencement of experiments (Uhlemann and Neims, 1972; Jeong et al., 2011). In our study, animals fasted for 12 h and behavioral testing commenced after 3 weeks of their being fed a KD. We also reported that animals were no longer in a state of established ketosis 1 h after the introduction of the normal pellet diet, indicating that the sustainability of ketosis is dependent on dedicated, timely feeding of the KD. In this work, we ensured that all animals were in states of ketosis before neural injury. However, when animals first enter ketosis, there are spikes in adenosine levels, which could have beneficial short-term effects (Yang et al., 2017). Future studies, with careful monitoring of the state of ketosis, could introduce the diet after nerve injuries to test the therapeutic potential of a KD in alleviating the maintenance of neuropathic pain and motor disability.

Mirroring this behavioral observation are our molecular findings showing no changes in the levels of neuromodulators after the commencement of KD. We chose to study the levels of dopamine, serotonin, and noradrenaline as they share common mechanisms underlying pain and motor functions (Hains et al., 2001; Scott et al., 2006; Rommelfanger et al., 2007). Their levels were determined in the spinal cord, which is a proxy measure of monoamine release at the terminals rather than cell bodies originating from the brain. Nonetheless, the pathophysiology of pain and motor disabilities is complex, and systemic administration of KD is likely to affect multiple downstream signaling pathways despite the monoamine systems. Of particular interest is the expression of brain-derived neurotrophic factors (Keefe et al., 2017) and other inflammatory mediators, which may be modulated by KD (Vizuete et al., 2013) and have an established role in nerve repair (Zhang et al., 2000), plasticity (Seebach et al., 1999), and pain (Coull et al., 2005). Moreover, KD can decrease oxidative stress following traumatic brain injury (Greco et al., 2016). It was found by Streijger et al. (2013) that KD upregulated glucose transporter 1 and monocarboxylate transporter-1, and it is possible that this also occurred in our work. In addition, our study detected a decrease in the levels of tyrosine and tryptophan from KD mice, which are substrates that are important for the production of monoamines (Wurtman and Fernstrom, 1975). A plausible explanation for the mismatch in levels of precursors and monoamines is the bioavailability of converting enzymes, including tyrosine hydroxylase controlled by changes in diets (DeCastro et al., 2005). Alternatively, the diets used have different concentrations of the amino acids tryptophan and tyrosine with the KD diet (catalog #3666, Bio-Serv), having lower amounts of both (Table 1). To complete the findings, further investigation of the expression of other amino acid precursors and converting enzymes would be beneficial.

In summary, our study represents the initial exploration into the therapeutic potential of KD in nerve injury-related disease and showed that KD might not be suitable as a therapy for pain and motor recovery for the hindlimb. A translational approach was used to study the effects of pain and motor recovery from sciatic nerve injury and T10–T11 SCI. Our outcome measures showed a minimal effect of the diet and provided a mechanistic insight describing no change in levels of monoamine release at spinal terminals. Other studies could consider a different direction to further elucidate the effect of KD, including a later initiation of the diet or a refocus on a different disease phenotype.

## Synthesis

Reviewing Editor: Eran Perlson, Tel Aviv University Decisions are customarily a result of the Reviewing Editor and the peer reviewers coming together and discussing their recommendations until a consensus is reached. When revisions are invited, a fact-based synthesis statement explaining their decision and outlining what is needed to prepare a revision will be listed below. The following reviewer(s) agreed to reveal their identity: Dianna Willis, Isobel Scarisbrick.

In this manuscript, the authors investigate the extent to which a ketogenic diet can improve sensorimotor behavior follow either a CNS (T10-11 hemisection SCI) or a PNS (sciatic nerve spared nerve injury) injury. Previous studies in other SCI models, as well as brain injury and diseases, have suggested that a ketogenic diet may be neuroprotective and/or analgesic through several putative mechanism. This is the first study to look at its effect on hindlimb function following a thoracic level injury. The authors present data that the ketogenic diet had no beneficial effect on sensorimotor behavior in either injury paradigm, even though the diet was sufficient to increase ketone levels. The diet also had no effect on the spinal cord levels of monoamines, which given their role as neuromodulators of locomotion and nociception, might be expected to change if the diet was acting via this mechanism. Interestingly, there was decreased tryptophan and tyrosine in the spinal cords of KD mice. Overall, this is a well-written manuscript describing a potential therapeutic approach that has received considerable interest given its utility in other pathological conditions and the relative ease of administering. I think it is important to publish studies that do not support the hypothesis, especially given this level of interest as a potential therapeutic approach.

However, mechanistic explanations for why results differ from other findings in the SCI field are not well addressed and there are still questions regarding timing of treatment, duration of treatment, outcome measures, etc that make it difficult to conclusively conclude that this is not a viable therapeutic for this type/location of injury.

Major Comments:

1. Could the changes in tryptophan and tyrosine simply be due to the differences in diet composition? It would be helpful to comment on the amounts of these in each of the diets. Wouldn’t you expect to see the opposite based on the components of a ketogenic diet? Is the tryptophan and tyrosine depletion a consequence of alterations in metabolic activity?

2. Body weights were not significantly lower by diet alone, yet there was a significant effect of the interaction (diet x time) in the mixed-effect analysis. Since time here is influenced by the surgery status, this should be commented on.

3. Why might mechanical allodynia be increased in the KD mice? Increased inflammation? Is it a sign of a more intact sensory systems or altered neuronal activity?

4. Although the authors do mention that forelimb recovery was improved in rodent models of SCI, they did not mention human pilot studies that show improved motor outcomes in upper extremities (i.e. Yarar-Fisher et al, Spinal Cord Ser Cases, 2018).

5. The authors have done an excellent job showing that functional outcomes are not improved by KD. However, since they only looked at behavior and a single tissue endpoint, it complicates the interpretation of the results being an assumption that no beneficial or detrimental effects are occurring at the tissue level (i.e. on inflammation, oxidative stress, metabolic changes). Do authors suggest that no underlying mechanism is changed, thus no functional changes? For example, inflammation could lead to increased tryptophan catabolism, yet no inflammation markers are looked at. This should be addressed.

6. It is acknowledged that the type of injury/location is different from other reported studies, but are the expected underlying mechanisms for cited literature simply not affected by KD in this model? If so, why might this region of spinal cord not have the beneficial effect when compared to cervical? It is interesting that the SCI and peripheral nerve would both not show changes here, yet another SCI model showed benefit. Would this indicate that the diet itself (both are ketogenic but might have a differences in composition), rather than anatomical location, could have an effect?

7. Overall, it looks like statistical methods are described appropriately, but why are there many more PD versus KD group mice? How were the animals assigned to the diet groups?

8. The last sentence in the Abstract seems to directly contradict the results shown. “This suggests that ketogenic diets need to be assessed based on the type and location of neurotrauma” comes directly after discussing that the diet had no effect in either the T10-11 injury or the SNI injury. Maybe you mean this statement in comparison to prior studies, but this is confusing and needs to be better explained if that is the point you are trying to make.

9. The authors talk in the discussion about the timescale of the post-injury behavior in their study vs. the published cervical study. In looking at those data, pellet reaching was not significant until at least 6 weeks, although cylinder reaching was at 2 weeks. How convinced are the authors that they looked out late enough, or that they used the best behavioral outcomes, to see any potential effect. Have they considered additional outcome measures, such as CatWalk, where you can get more detailed aspects of locomotion such as gait pattern and sensory-motor control? I’m not so sure that the difference in effect vs. non-effective in the prior published study and this study is largely due to injury type/location.

10. The heading “The ketogenic diet had a moderate effect on sensorimotor recovery in the context of peripheral nerve injury” is not consistent with the data shown. There is no improvement in recovery in either model. This is misleading.

11. Figure 2 demonstrates no changes in monoamines. Where any other measures of “plasticity” measured (such as HIF, BDNF, etc)

Minor Comments:

1. Can you provide additional details on randomization? Was baseline behavior done prior to diet randomization, so that groups were balanced based on baseline behavior?

2. In Figure 3I, the authors talk about a “trend”. The difference is not significant.

3. Period instead of comma on page 12, first paragraph, between “behavioral outcomes” and “object recognition"

4. Page 12, second paragraph. Comma after “peripheral nerve injury”.
